# Finite Element Modeling of Microstructures in Composite Materials: A Special Issue in *Materials*

**DOI:** 10.3390/ma16155332

**Published:** 2023-07-29

**Authors:** Yunhua Luo

**Affiliations:** Department of Mechanical Engineering, University of Manitoba, Winnipeg, MB R3T 5V6, Canada; yunhua.luo@umanitoba.ca

This Special Issue of the journal Materials aims to gather recent advancements and novel developments in the field of finite element modeling of microstructures in composite materials. The primary focus of this Special Issue is to explore the intricate relationship between composite microstructure and its macroscopic behavior through the utilization of finite element modeling techniques. By showcasing the latest research, this Special Issue aims to enhance our understanding of the fundamental principles governing the mechanical response of composites at the microscale and their implications at the macroscale. Researchers from various disciplines are encouraged to contribute their valuable insights, methodologies, and findings, thus fostering a collaborative environment for advancing the field of microstructural modeling in composite materials.

The microstructure of composite materials is incredibly intricate, encompassing the arrangement, distribution, and interaction of constituent components, such as phase materials, voids, and defects. It plays a crucial role in determining the macroscopic behavior of composites, and the relationship between microstructure and macroscopic behavior is highly complex. To investigate this relationship, various tools have been employed, including analytical analysis, mechanical testing, and numerical modeling. Among these, numerical modeling, particularly finite element simulation, offers numerous advantages. The development and refinement of finite element models for microstructures in composite materials have revolutionized virtual testing and prototyping. This approach significantly reduces the reliance on expensive and time-consuming experimental trials, making it a cost-effective and efficient tool for exploring different design configurations and optimizing composite structures. Finite element modeling excels in predicting and analyzing the failure mechanisms of composite materials. By simulating crack initiation and propagation, delamination, and other damage mechanisms, researchers can pinpoint critical regions, evaluate the structural integrity of the material, and propose strategies to enhance its durability and reliability. Leveraging finite element modeling empowers researchers and engineers to gain valuable insights into the effects of microstructure on composite behavior, enabling informed decision-making in composite material design and optimization processes.

In terms of future implications, the advancements in finite element modeling of microstructures in composite materials hold immense potential for further progress. Continued developments in computational techniques, algorithms, and multiscale modeling approaches will contribute to even more accurate and efficient simulations. The integration of experimental data with modeling results will synergistically enhance predictive capabilities, resulting in more reliable and optimized composite materials.

Moreover, the integration of finite element modeling with artificial intelligence and machine learning techniques opens up exciting opportunities for data-driven material design and optimization. The ability to rapidly analyze vast amounts of data, extract meaningful patterns, and generate novel microstructures with desired properties will revolutionize the field, accelerating the development of advanced composite materials.

In summary, the field of finite element modeling of microstructures in composite materials is of paramount importance with far-reaching implications. Its impact extends from fundamental research to industrial applications, enabling improved material design, enhanced performance, and cost-effective solutions. The ongoing advancements in this area will undoubtedly shape the future of composite materials, driving innovation, and pushing the boundaries of their capabilities.

